# The outcomes of chemotherapy only treatment on mild spinal tuberculosis

**DOI:** 10.1186/s13018-016-0385-y

**Published:** 2016-05-14

**Authors:** Zehua Zhang, Fei Luo, Qiang Zhou, Fei Dai, Dong Sun, Jianzhong Xu

**Affiliations:** Department of Orthopaedics, Southwest Hospital, Third Military Medical University, Chongqing, China

**Keywords:** Mild spinal tuberculosis, Standard chemotherapy alone, Treatment outcome

## Abstract

**Background:**

The treatments for spinal tuberculosis (TB) patients without absolute surgical indications have been controversial. Some people believed that most spinal TB patients were indicated for surgery, while other people believed in chemotherapy only. To help clarify the treatment over spinal TB patients without absolute surgical indications, we characterized a subtype spinal TB and then analyzed the treatment outcomes of standard chemotherapy alone.

**Methods:**

In this retrospective study, 740 adult patients of spinal TB from January 2005 to January 2013 in our institution were reviewed. Patients who fit into the characterizations of mild spinal TB were started by standard chemotherapy for 18 months and followed up for at least 2 years upon the completion of treatment. The overall outcome, neurological function, local kyphosis, and level of pain at different time points were assessed.

**Results:**

After starting the conservative treatment, 89 out of 740 patients were chosen for chemotherapy alone, and all patients were followed up for at least 2 years (ranging from 24 to 50 months) upon the completion of the treatment. Of 89 patients, 95.4 % of patients showed a definite and clinical response within 1 month after starting the treatment, 69 % of patients had excellent to good results, with no complications of the disease, and 77.5 % had asymptomatic local kyphosis with intact neurological function; solid bony fusion of adjacent segment was achieved in 88.8 % of patients.

**Conclusions:**

We believe that the mild spinal TB respond well to the standard chemotherapy, and the detailed description of mild TB would provide crucial guidance in determination of conservative treatment.

## Background

The World Health Organization reports that China ranks second among the 22 countries with the highest burden of tuberculosis [1]. Spinal tuberculosis (TB) is the most common form of extrapulmonary tuberculosis, accounting for approximately 1 to 3 % of all tuberculosis cases [2].

The management for spinal TB includes conservative and operative treatments. So far, the absolute surgical indications for spinal TB include (1) progressive neurologic deficit, (2) progressive increase in spinal deformity (coronal or sagittal) (3) failed conservative treatment including 1 and 2 above or severe pain due to abscess or spinal instability, and (4) uncertain diagnosis: this could be an inability to obtain microbiological diagnosis from microscopy, culture, or even via detection of mycobacterium DNA using polymerase chain reaction (PCR) techniques [3]. Meanwhile, there is no agreement upon the indications of conservative treatment. Several classifications have been proposed to help with the determination, and most spinal TB patients were recommended for surgery accordingly. On the other hand, however, recent literatures questioned the efficacy of surgery in improving the outcomes of spinal TB [4].

Fundamentally, the treatment of tuberculosis is by chemotherapy and surgery attempts only to extirpate the complications arising from the disease process. Hence, most cases of spinal TB merit medical rather than surgical treatment, especially the mild spinal TB.

In the present study, we sought to clarify the indications of conservative treatment for spinal TB by analyzing the outcomes of mild spinal TB cases treated by nonoperative methods in our center.

## Methods

A retrospective study was made of 740 consecutive adult patients with spinal TB who were diagnosed and treated in our center from January 2005 to January 2013. The diagnosis of spinal TB was made upon the medical history, clinical manifestations, radiology, lab tests, and histology samples if possible. Only patients who started conservative treatment when they were presented to our clinic were included in our study. The indications of conservative treatment included (1) mild to moderate neurologic deficit (no less than American Spinal Injury Association (ASIA) D), (2) single vertebral involvement with central lesion or multivertebral involvement (less than 3) with edge type lesion, (3) vertebral accessory tuberculosis without spinal canal involvement, (4) vertebral body collapse less than 1/3 of total height, (5) limited range of paravertebral abscess (within one level vertebrae, no retropharyngeal abscess, or psoas abscess), (6) no significant kyphosis (less than 30°), and (7) no significant intervertebral instability [5] (a sagittal plane displacement greater than 2.5 mm in the thoracic spine as demonstrated on lateral radiographs was considered potentially unstable, a sagittal plane displacement greater than 4.5 mm in the lumbar spine, or 15 % of the anteroposterior diameter of the vertebral body as demonstrated on a static lateral radiograph, was considered potentially unstable). The contraindications for conservative treatments included skeletal immaturity, pregnancy at the time of treatment, intracanal tumor or metastatic tumor, poor compliance to treatment due to mental conditions, and significantly impaired hepatic function (alanine aminotransferase, ALT and aspartate aminotransferase, AST higher than two times of normal level).

Patients who met all the above criteria were treated conservatively, and patients with deteriorating neurologic function (worse than ASIA D) or progressive kyphosis (greater than 40°) while on anti-tuberculosis chemotherapy were considered for surgical treatments. Approval from our institutional review board was obtained for the study. A written informed consent was obtained from each of the patients authorizing treatment, radiographic examination, and photographic documentation and publishing the above data in academic studies.

Detailed medical history and physical examination were acquired for each patient; neurological status was evaluated at each follow-up visit. Muscle power was examined and scored from 0 to 5 according to the Medical Research Council scale. Spinal cord function was evaluated using ASIA definitions. Muscle tone, reflexes, pinprick sensations, joint position, and vibration were tested, and the presence of bone deformity and soft-tissue tenderness or swelling was also recorded. Radiology including plain X-rays, CT, and MR was used to evaluate spinal features such as osteopenia, paravertebral abscess, disc space reduction, and endplate erosion, as well as gross vertebral and costovertebral bone destruction and deformities. In addition, the following lab tests were done for each patient in the first visit: complete blood count, erythrocyte sedimentation rate (ESR), liver function, and HIV serology. Liver function and ESR were monitored at each follow-up after the drug administration. Localized spinal deformity was measured as the angle between the upper and lower endplates of the collapsed vertebral levels using the Cobb method. Radiological evaluation was made before treatment and at each follow-up visit.

### Treatment protocol

1. Chemotherapeutic agents were administered using a standard protocol. Patients were treated with a standard four-drug treatment that included rifampicin (15 mg/kg; maximum 600 mg/day), isoniazid (5 mg/kg; maximum 300 mg/day), pyrazinamide (25 mg/kg; maximum 2 g/day), and ethambutol (15–25 mg/kg; maximum 2 g/day)for 18 months [6].
2. Plain X-rays and MRI scans/CT scans and blood tests, as mentioned previously, were performed at 1, 3, 6, 9, 12, 18, 24, 30, and 42 months after starting the treatment to monitor response to treatment.
3. Patients who were ambulatory at the time of diagnosis were kept ambulatory during treatment, with restriction of heavy, load-bearing, contact activities. Patients who were bedridden at the time of diagnosis were kept in bed until clinical recovery (pain relief and neurological improvement) and then mobilized and ambulated with similar precautions as the ambulatory group, that is, with restrictions in heavy activity. Ambulation was permitted in these patients at an average of 3 to 4 weeks, and return to activities of daily living, light work, and noncontact activity was possible at 6 to 12 weeks, depending on the clinical response to treatment.
4. Information regarding treatment risks, such as death and morbidity, and the risks of chemotherapeutic management was provided to the patients. Patients were followed up at 1, 3, 6, 9, 12, 18, 24, 30, and 42 months after starting the therapy. Patients’ families were also given education about the importance of drug administration according to the protocol and adverse events related to the chemo agents [6].

### Ethics

This study was approved by the Institutional Review Board (IRB) of Southwest Hospital, Third Military Medical University. All procedures in the study were in accordance with the ethical standards of the IRB of Southwest Hospital and with the Helsinki Declaration.

## Results

After starting the conservative treatment, 89 out of 740 patients were chosen for chemotherapy alone, and all patients were followed up for at least 2 years (ranging from 24 to 50 months) upon the completion of the treatment. Of the 89 patients, 34 (52 %) of the patients were female and 55(48 %) were male, with a mean age of 28.08 years (range 15–78 years). Two patients were treated by standard anti-tuberculosis treatment protocol because of pulmonary TB for 24 months, and they had fully recovered for at least 5 years before being presented to our center due to spinal TB. Symptoms reported in the first visit included back pain, sweating, and fever, which were summarized in the table (Table 1). The involved spinal locations were the cervical spine in 2 patients (2.2 %), the thoracic spine in 21 (23.6 %), the thoracolumbar junction in 14 (15.7 %), the lumbar spine in 50 (56.2 %), and the sacral region in 2 (2.2 %). The most commonly affected vertebra was L4 in 23 cases (25.8 %), followed by L3 in 15 cases (16.9 %). Of all patients, 81 (91 %) had intervertebral destruction, 56 (62.92 %) had prevertebral or paravertebral abscesses, and 69 (77.53 %) had significant local kyphosis due to the collapse of the anterior column. No patients had epidural compression or spinal cord abscess. Of the cases with abscess, 25 (28.09 %) were larger than 6 cm and 22 (24.72 %) were larger than 4 cm in diameter. As indicated on MR images, a single vertebra was involved in five patients (5.62 %), two vertebrae in 78 (87.64 %), and three vertebra in 6 (6.74 %) patients. No patient had pulmonary or lymph node tuberculosis.

**Table 1:** The main symptoms, radiological data and lab results of patietns in the first visit

	Variable	Value (%)
Sex		
	Male	34 (52)
	Female	55 (48)
Age (years)		
	15–40	43 (48.3)
	41–60	35 (39.3)
	>60	11 (12.4)
Symptoms at presentation		
	Pain	87 (97.75)
	Night sweat	33 (37.08)
	Low temp fever	25 (28.09)
	Marasmus	39 (43.82)
Physical signs		
	Restriction	80 (89.89)
	Percussion pain	88 (98.88)
	Local deformity	10 (11.24)
	Sinus	2 (2.25)
ESR (mm/h)		42.35 ± 6.31
Drug-resistant tests		31
	Sensitive	15 (48.39)
	Resistant	4 (12.09)
Numbers of involved vertebra		
	1	5 (5.62)
	2	78 (87.64)
	3	6 (6.74)
Imaging appearance		
	Disc space destruction	81 (91.01)
	Paravertebral abscess	56 (62.92)
	Local kyphosis	69 (77.53)
	Vertebral body destruction	84 (94.38)
Affected spinal level		
	Cervical	2 (2.2)
	Thoracic	21 (23.6)
	Thoracolumbar	14 (15.7)
	Lumbar	50 (56.2)
	Sacral	2 (2.2)
Neurological signs		
	Pretreatment (ASIA D)	11 (12.4)
	Posttreatment (ASIA D)	6 (6.7)
	Pretreatment (ASIA E)	78 (87.6)
	Posttreatment (ASIA E)	83 (93.3)
Kyphosis (°)		
	Pretreatment	6.2 ± 3.11°
	Posttreatment	14.36 ± 6.31°
Mean VAS score		
	Pretreatment	7.6
	Posttreatment (12M)	1.7
	Posttreatment (24M)	0

After beginning conservative medical management, 85 (95.4 %) of the 89 patients showed definite and persistent clinical response within 1 month, which was confirmed by radiological findings and blood tests. One year after the completion of treatment, the mean VAS score had improved from a pretreatment score of 7.6 to a posttreatment score of 1.7 (*p* < 0.001, paired sample *t* test), accompanied by complete resolution of pretreatment symptoms. Two years after the completion of treatment, the average VAS score was 0. Muscle strength was normal in 78 patients (91.76 %) with an ASIA grade of E, but hyperflaxia of tendon reflexes were present in some of these patients.

Four patients (4.5 %) did not respond to the four first-line drugs in the first month, and in the third month follow-up, they complained of severe back pain (average VAS 8.5); they also exhibited neurological deficits which were given an ASIA grade of D. The radiology showed significant progression of the vertebral destruction and paraverterbal abscess. Those patients were then treated with surgical correction, bone grafting, and chemotherapy with second-line drugs. Consequently, the outcomes of the four patients were not included in the following analysis.

**Fig. 1:**
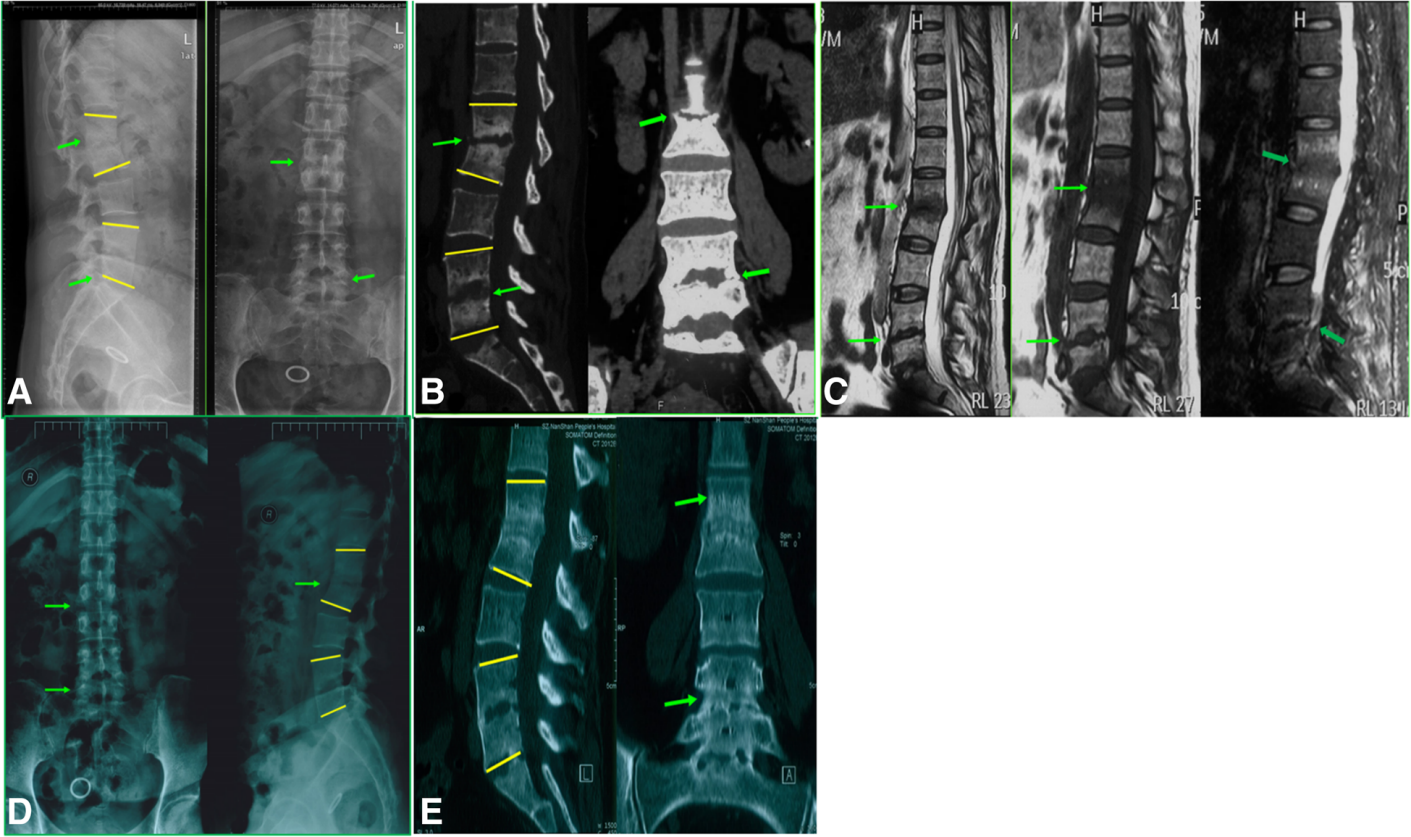
These are radiology data for a 44-year-old female with mild spinal tuberculosis at L1/L2 and L4/L5. The patient was treated with standard first-line antituberculosis drugs for 18 months. **a** The lateral view of X-ray shows that, before the treatment, there was bone destruction between the L1/L2 and L4/L5 vertebral (*arrow*) and the Cobb angles (*yellow lines*) for the local kyphosis were 20.3° and 10.5°, respectively. The anterior and posterior views show significant intervertebral space reduction (*arrow*) of L1/L2 and L4/L5. **b** The CT images before the treatment show bone destruction and intervertebral space reduction (*arrows*) at L1/L2 and L4/L5 levels, and the Cobb angles (*yellow lines*) of kyphosis were 22.6° and 8.5°, respectively. **c** The MRI images before the treatment show significant edema and absence of normal intervertebral signal at L1/L2 and L4/L5 level (*arrows*), but there is no compression on the spinal cord or nerve roots. **d** The anterior posterior and lateral views of X-ray show that, after 18 months of standard chemotherapy of first-line drugs, there is no progression of bone destruction or vertebral collapse, and there is formation of bonny bridging (*arrows*) between the L1 and L2 vertebra, which indicated possible simultaneous spinal fusion. The Cobb angles of local kyphosis (*yellow lines*) were 20.8° and 11.3°, respectively. **e** Two years after the 18-month standard chemotherapy treatment, the CT images show solid fusion at the L1/L2 and l4/L5 levels, and the Cobb angles of local kyphosis (*yellow lines*) were 19.8°and 9.8°, respectively. There was no significant progression of local kyphosis compared to those of pretreatment

**Fig. 2:**
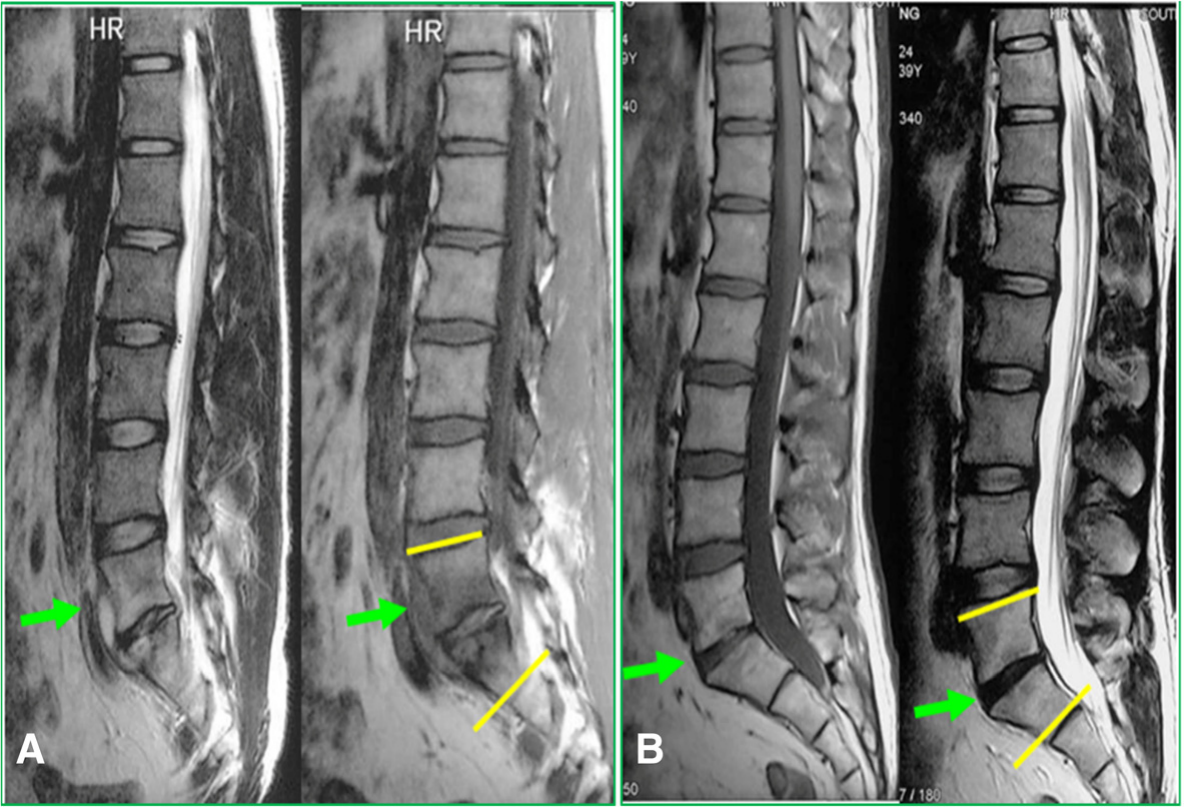
This is a 34-year-old male who was diagnosed of mild spinal tuberculosis (L5/S1) with sacral abscess; the patient was treated with standard chemotherapy for 18 months. **a** The MRI before the treatment shows an intervertebral edema of L5/S1 and the formation of sacral abscess anteriorly (*arrow*); the Cobb angle of local lordosis (*yellow lines*) was 33.4°. **b** The MRI after 5 years shows complete absence of sacral abscess and the intervertebral edema, and the Cobb angle of local lordosis (*yellow lines*) was 31.5°; there was no significant progression of local kyphosis or simultaneous spinal fusion

The Cobb angles of the local kyphosis before and 2 years after completion of treatment were compared. The average angulations were found to have increased significantly from 6.25 ± 3.11°(range 10°–32°) to 12.36 ± 6.31°(*p* < 0.05, paired sample *t* test). A regression of spondylodiscitis and abscess absorption was a sign of recovery as demonstrated on MR images; nine cases (10.59 %) showed total recovery of vertebral body and vertebral disc according to the radiological image (Fig. 1.). Seventy-nine (87.05 %) showed spontaneous spinal fusion between the destructed vertebral and adjacent levels; 69 (81.18 %) had asymptomatic local kyphosis with intact neurological function (Fig. 2). As to the adverse effects of chemotherapy, 11 (12.94 %) had transient gastrointestinal reaction (nausea and vomiting) after the first week of chemotherapy, which then resolved after medications of antiemetics. Five cases (5.88 %) showed elevated ALT (three times higher than normal level) and AST (two times higher than normal level) in one visit and went back to normal after hepatoprotective treatment. There were no reports of optic nerve or auditory nerve injury due to the chemotherapy.

Finally, according to the results above, we modified a previous scoring system [7] and classified the outcomes into four categories by considering the following criteria:

1. Normal body temperature and ESR, negative sign of all pretreatment symptoms
2. No presence of sinus or abscess
3. Intact neurological function
4. Complete regression or calcification of lesion focus on CT/MRI scan
5. Primary-line four-drug anti-tuberculosis medications, used throughout the 18 months
6. More than four drugs used, for longer than 18 months
7. Progression of kyphosis over 10° at affected level at the end of treatment
8. Failure of nonoperative treatment, requiring surgical intervention

The four categories are as follows:

- Excellent result: complete resolution of disease with first-line anti-tuberculosis treatment for 18 months, with no residues or side effects of treatment (criteria 1, 2, 3, 4, 5)
- Good result: complete resolution of disease with first-line treatment for longer than 18 months, or with second-line drugs for 12 months or longer, with or without the occurrence of medically manageable side effects of drug therapy, such as gastrointestinal intolerance and drug-induced jaundice, necessitating frequent manipulations in their doses and regimen, but with eventual complete resolution (criteria 1, 2, 4, 6)
- Fair result: complete resolution of disease, but with increased kyphosis of 10° or more at the affected level with no obvious or mild neurological compromising (criteria 1, 2, 3/4/5/6, 7)
- Poor result: cases that did not respond to conservative treatment, primary or secondary line, and had to be eventually operated (criteria 8)

The results of our series of 89 cases are shown in Table 2. Thus, excellent to good results were seen in over 69.66 % of our patients, with nonsurgical treatment. The 25.84 % with fair results also resumed routine activities of work and daily living without physical restrictions or need for regular analgesic medications, despite residual kyphosis. Four patients (4.49 %) who were treated surgically were classified as poor.

**Table 2:** The outcomes of our series of 89 cases

	Number of cases (total = 89)	Percentage (%)
Result		
Excellent	20	22.47
Good	42	47.19
Fair	23	25.84
Poor	4	4.49

## Discussion

The World Health Organization reports that China ranks second among the 22 countries with the highest burden of tuberculosis; spinal TB is the most common form of extrapulmonary tuberculosis and remains a severe public health threat in China [1].

The indication of conservative treatment for spinal TB is a continuing matter of debate. Historically, the chemotherapy was considered as the only effective way of treating spinal TB, until Hodgsonet al. [8] advocated progressive surgical intervention. Later on, to solve the debate upon the treatment choice, the British Medical Research Council (BMRC) have initiated a series of clinical trials since 1970, the trial included two branches, the Korea and Hongkong branches. In Korea, 350 patients of spinal TB were enrolled for the medical management, and in Hongkong, 150 patients all underwent surgery. Though the disease was more extensive in Korea, the great majority of patients in both countries achieved a favorable status at 15-year follow-up [9]. Yet, on finishing the trial, they concluded that the surgical group had best results because these patients had more rapid abscess resolution and earlier and more frequent bone fusion [10]. Obviously, the lack of universal inclusion criteria and sample balance of trial branches have weakened the credibility of this conclusion. Recently, Chandra et al. described their paradigms of management, in which they claimed that radical, instrumented surgeries should be offered in an early stage [11]. Though the surgical intervention may have several advantages [3, 4, 12–14], it requires expertise, good anesthesia, and excellent perioperative care. It also requires hospitalization and is expensive and potentially dangerous. Complications can occur during the operation or postoperatively, which include reconstruction-related, vascular, neurological, visceral, and wound-related complications [3, 15–18]. Reconstruction failures can be breakage of the graft, screws, and rods, loss of correction, and failure of fusion [19]. Vascular problems during surgery can be massive bleeding, hematoma formation, and thromboembolism. Neurological damage of surgery can be nerve root lesion, dura tears, spinal cord infarction, and plexus lesions. Visceral damage, especially ureteric lesions, can occur. Wound infections happen in 1 to 6 % of spinal surgeries [17].

Meanwhile, Tuli et al. [20] believed a combined method in treating the spinal TB and they advocated a “middle path” regimen, which included a less radical surgery along with chemotherapy, but they did not come up with a clear cut protocol to distinguish the surgical candidates from those who do not need surgeries.

As a result, several classifications were proposed for clinical decisions. Mehta and Bohjraj [21] proposed a system based on MRI appearance; however, they did not have a subtype for conservative treatment. Oguz et al. [22] came up with a classification based on the size and location of the lesion, in which they believed only patients with one level vertebral involvement, no neurological deficit, no collapse, and no abscess could be treated by drug therapy alone. Unfortunately, some recent studies have proved that patients with larger lesions and certain degrees of neurological deficit could be successfully treated with nonoperative methods. Nene and Bhojraj [7] showed in their study that 98 % in their series could be treated conservatively. Kotil et al. [23] did a prospective study on patients without major neurological deficits or severe spinal deformities (kyphotic angles smaller than 30°). Forty-two (95.4 %) of the 44 patients were successfully treated with conservative medical management and attained acceptable spinal deformity angles. In a prospective study, Ahmed Bakhsh [6] included 26 patients with spinal TB who were followed up for 12 months. Eighty-five percent of cases completely improved on medical treatment without any surgical intervention. Nene and Bhojraj [7] assessed the efficacy and results of nonsurgical treatment in thoracic spinal TB in adult patients in a retrospective analysis of 70 adults with thoracic spinal TB. Over 98 % of the patients (69 of 70) were successfully treated conservatively. Seventy-four percent had excellent to good results, with no mechanical residues of the disease.

To clarify the indications for conservative treatment, we characterized a subtype spinal TB, which were most likely to benefit from medical treatment. Furthermore, we have come up with an all-inclusive evaluation system to verify the efficacy of medical treatment alone.

In our study, we characterized a subtype spinal TB, which is the “mild spinal TB” in great detail and selected the study population accordingly. As shown in Table 1, the enrolled patients were all skeletal matured and had less than 30° kyphosis with mild to moderate neurological deficit. As to the location of lesion, the most commonly affected levels were lumbar segments (56.2 %); the least affected were sacral and cervical. This might have to do with the neurological status at presence; as in the lumbar region, the nerve roots are more tolerant to the spinal deformity or vertebral collapse, while the cervical segments are more sensitive and display worse neurological deficit. This was unlike the previous studies, in which thoracic or thoracic lumbar were the most commonly involved segments [6, 11, 19, 23–26]. Deformity or kyphosis is a significant problem in Pott’s disease. Kotil et al. [23] reported that the mean local spine deformity angle had increased from 11° (range 5°–19°) before medical treatment to 21° (range 10°–32°) after treatment. Nene and Bhojraj [7] found that the progression of local kyphosis ranged from 0° to 40° at the 2-year follow-up, though the initial thoracic kyphosis of all patients was less than 30°. In the BMRC trail [9], the authors found the progression between 8° and 16° at 36-month follow-up. In our results, however, after 18 months of standard chemotherapy, the mean local kyphosis had increased from 6.25 at first visit to 14.36 at 24-month follow-up, which was less than the progression in the literatures. Rajasekaran et al. [27] reported that the progress of deformity occurs in two distinct phases: phase I includes the changes in the active phase, and phase II includes changes after the disease is cured. The deformity progression is influenced by the severity of the angle before treatment, the level of the lesion, and the age of the patient. Therefore, the limited progression in our study could be explained by the following reasons: first, all the patients had a mild initial kyphosis and they were all skeletal mature. Furthermore, the long-term standard chemotherapy has been carried out effectively to ensure the elimination of the lesion. And last but not least, most involved vertebra were lumbar segments, which was unlike the previous literatures in which thoracic lumbar segments were most commonly involved [6, 11, 19, 23–26], this also indicated that patients with lumbar involvement tend to have less kyphotic progression. Different degrees of neurological improvements were reported in the literature [7, 24, 28–30]. Yet in our study, we did not see dramatic improvement of the neurological status for most patients at the end of 2-year follow-up. The reason could be because we only included patients with mild to moderate neurological deficit (ASIA D and E) and there were no patients with any intracanal abscess or spinal cord involvement. It is believed that the back pain could be caused by the compression of the paraverterbal abscess; therefore, the resolution of the perivertebral abscess could lead to significant pain relief [7, 12, 29, 30]. And most patients in our study have achieved significant pain relief after 12 months of chemotherapy and continued to get better at the end of the 24-month follow-up, though there was no significant difference between the results of 12 and 24 months.

Our study is not without limitations. Though most patients responded to the standard chemotherapy, four of them showed progression of the neurological deficit and back pain, which later were proved to be infected by drug-resistant mycobacterium. The failure of these cases emphasized the importance of drug susceptibility tests in the early stage of treatment. In our center, we have implemented DNA microarray for detection of isoniazid (INH) and rifampicin (RMP) resistance strains, which proved to be an accurate and rapid method [31]. That is why all the failed cases were all before 2008, when we began to implement the DNA microarray as routine tests for spinal TB patients.

## Conclusions

The mild spinal TB could be treated by standard chemotherapy alone. Surgery should be reserved for cases which do not respond well to chemotherapy. If the angle of spinal deformity and neurological deficits are within acceptable limits, medical management alone is sufficient enough.
